# Supplementary material for: Continuous evaluation of denoising strategies in resting-state fMRI connectivity using fMRIPrep and Nilearn
Source: PLoS Comput Biol. 2024 Mar 18;20(3):e1011942. doi: 10.1371/journal.pcbi.1011942 (PMC10977879; doi:10.1371/journal.pcbi.1011942)
Supplement: S1 Text — (PDF) [file pcbi.1011942.s001.pdf]

S1: Literature review and details on fMRI confounds regression

Annex A. Common families of confound regressors

Mitigating the variance introduced by confounding fluctuations is necessary to gain meaningful measures of brain connectivity (Power et al., 2015). The most common method to minimize the impact of confounds is the use of linear regression (Friston et al., 1994) to regress out nuisance signals. These nuisance signals fall into one of three general categories commonly acknowledged in the literature: head motion, physiological noise (cardiac and respiratory), and instrumental noises from the MRI. The most common confound regressors are extracted following basic processing steps (i.e., motion correction, field unwarping, normalization, bias field correction, and brain extraction):

- Motion realignment measures capture head motion, a well-known source of disturbances in fMRI signals (Friston et al., 1996; Hajnal et al., 1994) which causes distance-dependent signal correlations and introduces systematic bias in group comparisons (Power et al., 2012; Satterthwaite et al., 2012; Van Dijk et al., 2012). Six rigid-body motion parameters (3 translations and 3 rotations) are typically estimated relative to a reference image and used as confound regressors (Friston et al., 1996).
- Non-grey matter tissue signals (such as *white matter* and *cerebrospinal fluid*) are unlikely to reflect neuronal activity and can be dominated by a mixture of motion and physiological artifacts (Fox et al., 2005). This type of signal is captured by averaging signals within anatomically-derived masks.
- The global signal is a confound regressor extracted from averaging signals within the *full brain* volume (Fox et al., 2005). The global signal clearly captures motion and physiological fluctuations, but is also sensitive to global neural activity, making it a controversial choice for regression (Power et al., 2017; Saad et al., 2012).
- Scrubbing (Power et al., 2012) is a volume censoring approach to remove high motion segments in which the framewise displacement (see Materials and Methods section Participant exclusion based on motion) exceeds some threshold. The scrubbing approach is applied alongside head motion parameters and tissue signal regressors.
- Temporal high-pass filtering accounts for low-frequency signal drifts introduced by *physiological and scanner noise sources*.

The family of motion, non-grey matter and global signal regressors can be further expanded using their first temporal derivatives and their quadratic (square) terms (Satterthwaite et al., 2013) to capture potential non-linear effects of these noise sources. Optimal denoising results often require full expansion of head motion parameters (both derivatives and squares).

Aside from regressors directly modeling noise derived from realignment measures or anatomical properties, other approaches capture the impact of motion and non-neuronal physiological activity through data-driven methods:

- The principal component-based method CompCor (Behzadi et al., 2007; Muschelli et al., 2014) extracts reduced components from white matter and cerebrospinal fluid masks to estimate non-neuronal activity.
- Independent component analysis-based methods estimate spatially independent components representing brain activity and noise. To identify the components related to head motion, researchers used a data-driven classifier (ICA-FIX (Salimi-Khorshidi et al., 2014)) or a pre-trained model (ICA-AROMA (Pruim, Mennes, van Rooij, et al., 2015)).

Despite the abundance of measures for non-neural signals, there is no one class of measures that can capture all known noise. A combination of nuisance regressors is thus needed to address the different sources of noise, and different common strategies have been put forth in the literature.

Annex B. Common denoising strategies

Researchers developed and proposed different strategies with the aim of improving denoising approaches. A denoising strategy typically involves the selection of a subset of nuisance regressors from the broad list presented above. Head motion combined with non-grey matter tissue signals is one of the most basic approaches (Fox et al., 2005). Scrubbing combines the basic approach above with volume censoring, which can be implemented either by removing time points entirely (Power et al., 2012) or by adding “spike” nuisance regressors (Lemieux et al., 2007; Siegel et al., 2014). Anatomical CompCor regressors are usually applied along with the basic head motion parameters (Muschelli et al., 2014). ICA-AROMA requires two steps of denoising: (i) a partial regression to remove variance associated with noise ICA-AROMA components inside a full ICA decomposition preceded by (ii) a linear regression including the basic average of white matter and the cerebrospinal fluid signals regressors (Pruim, Mennes, van Rooij, et al., 2015). As there exists multiple variants of most categories of confounds, e.g. degree of expansion in motion parameters, a large number of specific variants do exist for each strategy through combinatorial effects. To provide objective guidance to practitioners in their selection of an effective denoising strategy, comprehensive denoising benchmarks have emerged in the functional connectivity research literature.

Annex C. Evaluation of denoising strategies

Denoising benchmarks use several metrics to evaluate the effectiveness of denoising strategies on functional connectivity, commonly including loss of temporal degrees of freedom, residual motion effects, and impact on network properties, such as modularity. The ability of different strategies to remove confounds is heterogeneous (Ciric et al., 2017), and no single strategy dominates across all evaluation metrics. Several benchmarks still recommended ICA-AROMA due limited loss of degrees of freedom compared to scrubbing-based methods (Parkes et al., 2018), possibly in combination with global signal

regression for maximal reduction of motion artifacts (Burgess et al., 2016; Ciric et al., 2017). However, a denoising strategy can perform differently due to factors that strongly correlate with motion, such as psychiatric conditions, age, or the choice of subsequent analytical technique. For example, CompCor may only be maximally effective in low-motion data (Behzadi et al., 2007). Volume-censoring-based strategies are unsuitable for time series analysis where a uniform sampling of signals is required, such as most time-frequency analysis implementations. Researchers thus need to evaluate the best denoising strategy based on the available benchmarks, the profile of their data and their choice of analytical techniques.

The existing benchmarks have a few pitfalls that limit the integration to each researcher's unique needs. The benchmark research and denoising methods development are conducted on in-house preprocessing solutions with different datasets. From the research standpoint, this is not necessarily a pitfall as it showcases that workflows built upon different software following the general shared principle for preprocessing have converging conclusions. However, this is a problem for user adoption of the recommended strategy to their choice of preprocessing workflow. To correctly implement the best approach for their study, researchers need to understand the extensive literature and then construct the workflow. Another pitfall is that results in benchmarks are subject to the scope of datasets evaluated. As researchers use in-house tools and analysis code, there is no direct way to apply the suggested strategy or generate the benchmark statistics for evaluation on a new dataset. The denoising benchmark literature provides a good overview of the progress of methods in the field of resting state functional connectivity, but falls short in providing a reproducible path for the general community to adapt the results to modern solutions of preprocessing, such as fMRIPrep.

Annex D. Implementation of ICA-AROMA in fMRIPrep

In fMRIPrep, the whole brain global signal regressor and the estimated head-motion parameters were calculated on the output from their regular pipeline (i.e., before denoising), which is inconsistent with the original proposal (Pruim, Mennes, van Rooij, et al., 2015) and not suitable for combining with ICA-AROMA. Based on the original implementation, the GSR should be calculated from the time series after removing the variance of ICA-AROMA regressors (Pruim, Mennes, van Rooij, et al., 2015). Otherwise,There is a possibility that the global signal regressor reintroduced motion to the data (Lindquist et al., 2019).

Annex E. The denoising procedure described in mathematical operation

Here's the procedure performed in `nilearn.signal.clean` based on the options we used for denoising in the current benchmark.

Given a continuously sampled or non continuously sampled (scrubbed) time series, there would be a set of confounds sampled in the same manner. Let X be the matrix representing the confounds $size(n_{confounds}, n_{timepoints})$ and Y the matrix representing the extracted raw fMRI signal with a given atlas $size\ (n_{parcels}, n_{timepoints})$. The confounds are standardized into z-score denoted as X_z. For each confounds at each time point $X_{(c,\,t)}$:

$$X_{z(c,\,t)} = \frac{X_{c,\,t} - \mu(X_c)}{\sigma(X_c)}.$$

- $\mu(X_c)$: average of row c of X
- $\sigma(X_c)$: standard deviation of row c of X

We perform a linear regression using the confounds as regressors and signal as as follows:

$$Y = \beta X_z + \varepsilon \text{ (1)}.$$

- β : matrix $size\ (n_{parcels}, n_{confounds})$ of regression coefficients
- ε: error matrix $size\ (n_{parcels}, n_{timepoints})$, in the context of denoising this is the signal irrelevant to the confounds.

Hence the cleaned signal Z can be obtained through:

$$Z = Y - \widehat{\beta} X_z .$$

- $\widehat{\beta}$: the estimated regression coefficient of (1).

Finally the cleaned signal is standardized as z-score, independently for each parcel p:

$$Z_{z(p,t)} = \frac{Z_{(p,t)} - \mu(Z_p)}{\sigma(Z_p)}.$$

- $\mu(Z_p)$: average of row p from Z
- $\sigma(Z_p)$: standard deviation of row p from Z

Figures: Loss of temporal degrees of freedom broke down by subgroups

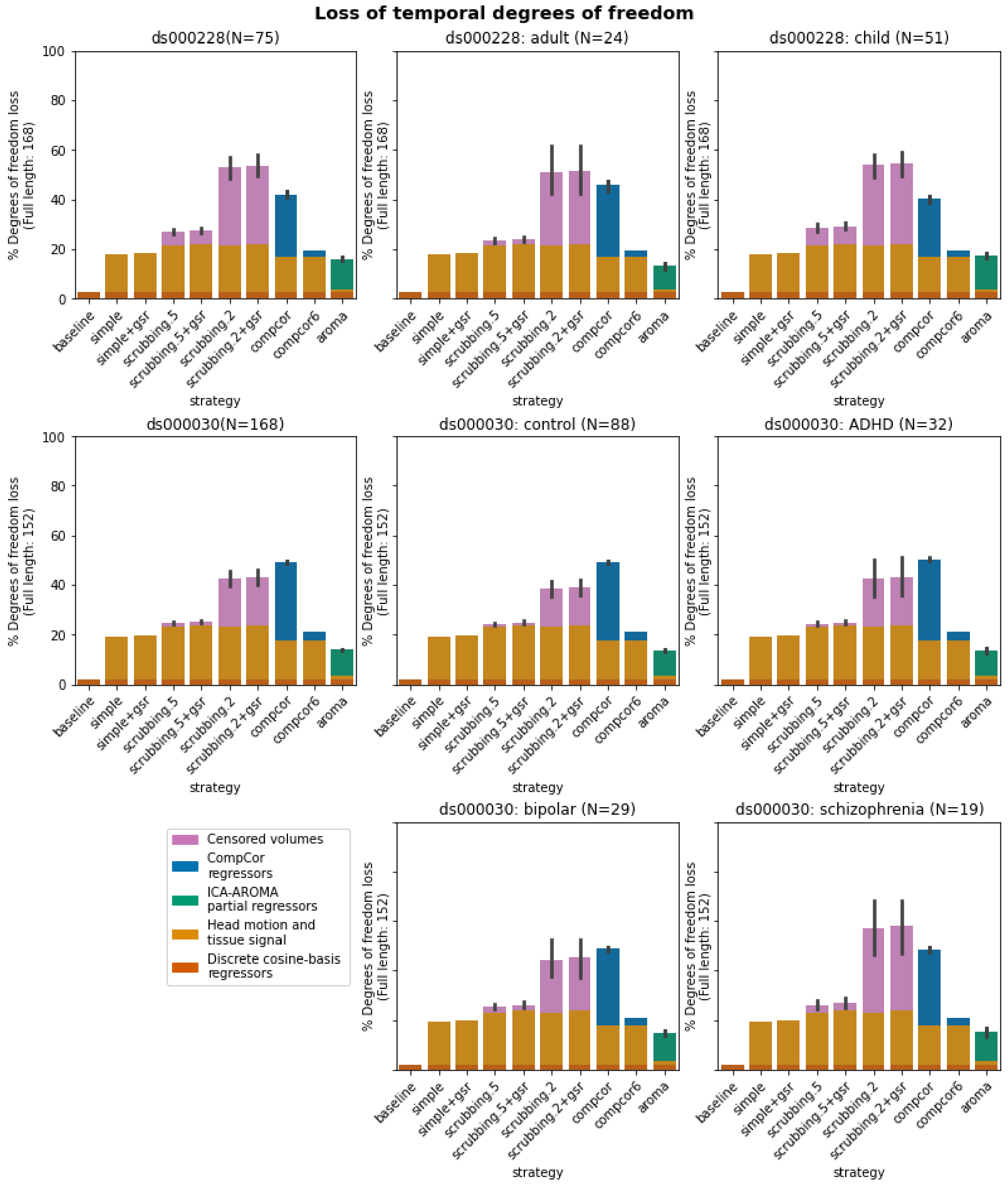

S1 Figure. Loss of temporal degrees of freedom broke down by subgroups after removing high motion subjects.

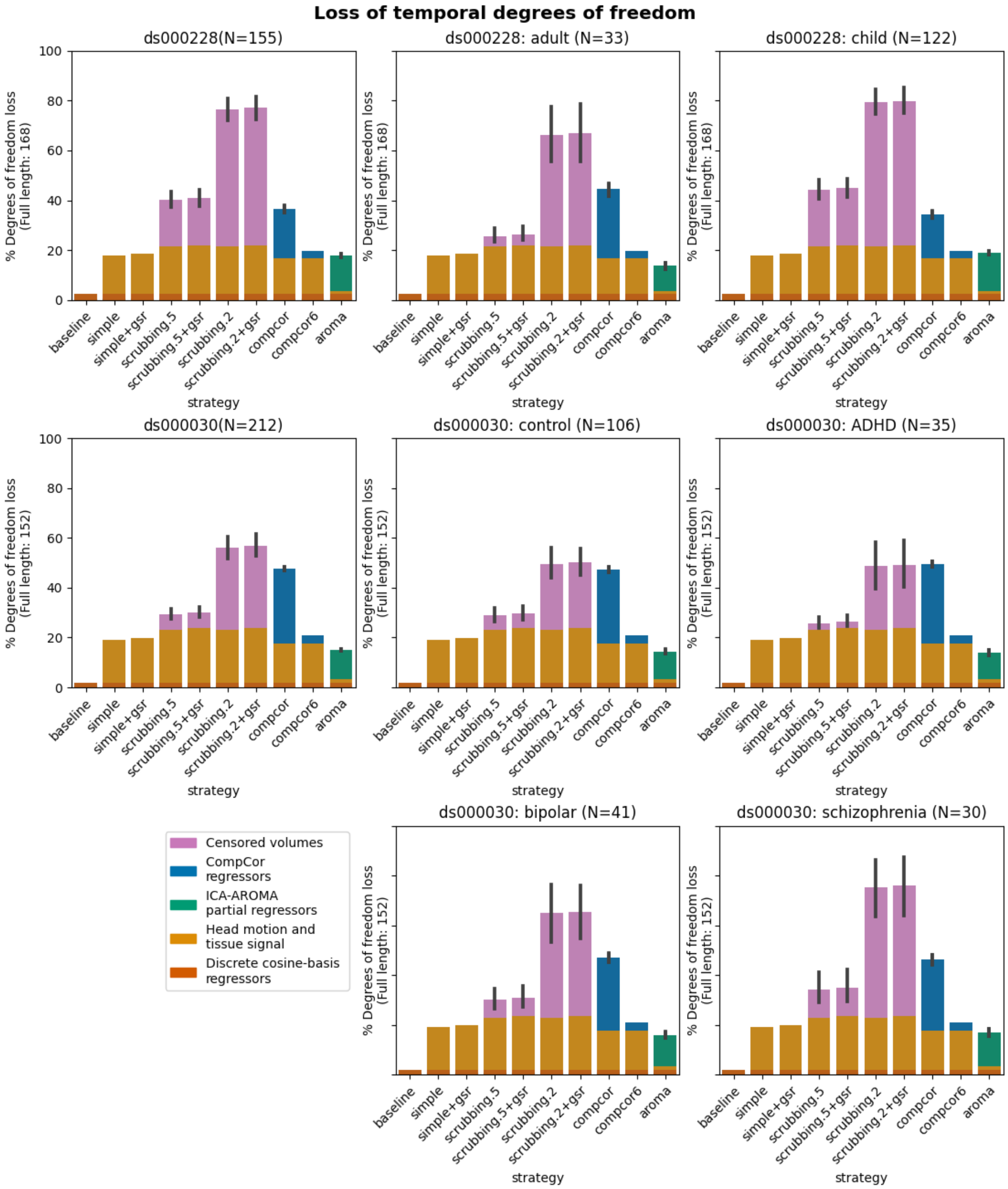

S2 Figure. Loss of temporal degrees of freedom broke down by subgroups before removing high motion subjects.
