# Supplementary figures and images for: Continuous evaluation of denoising strategies in resting-state fMRI connectivity using fMRIPrep and Nilearn
Source: PLoS Comput Biol. 2024 Mar 18;20(3):e1011942. doi: 10.1371/journal.pcbi.1011942 (PMC10977879; doi:10.1371/journal.pcbi.1011942)

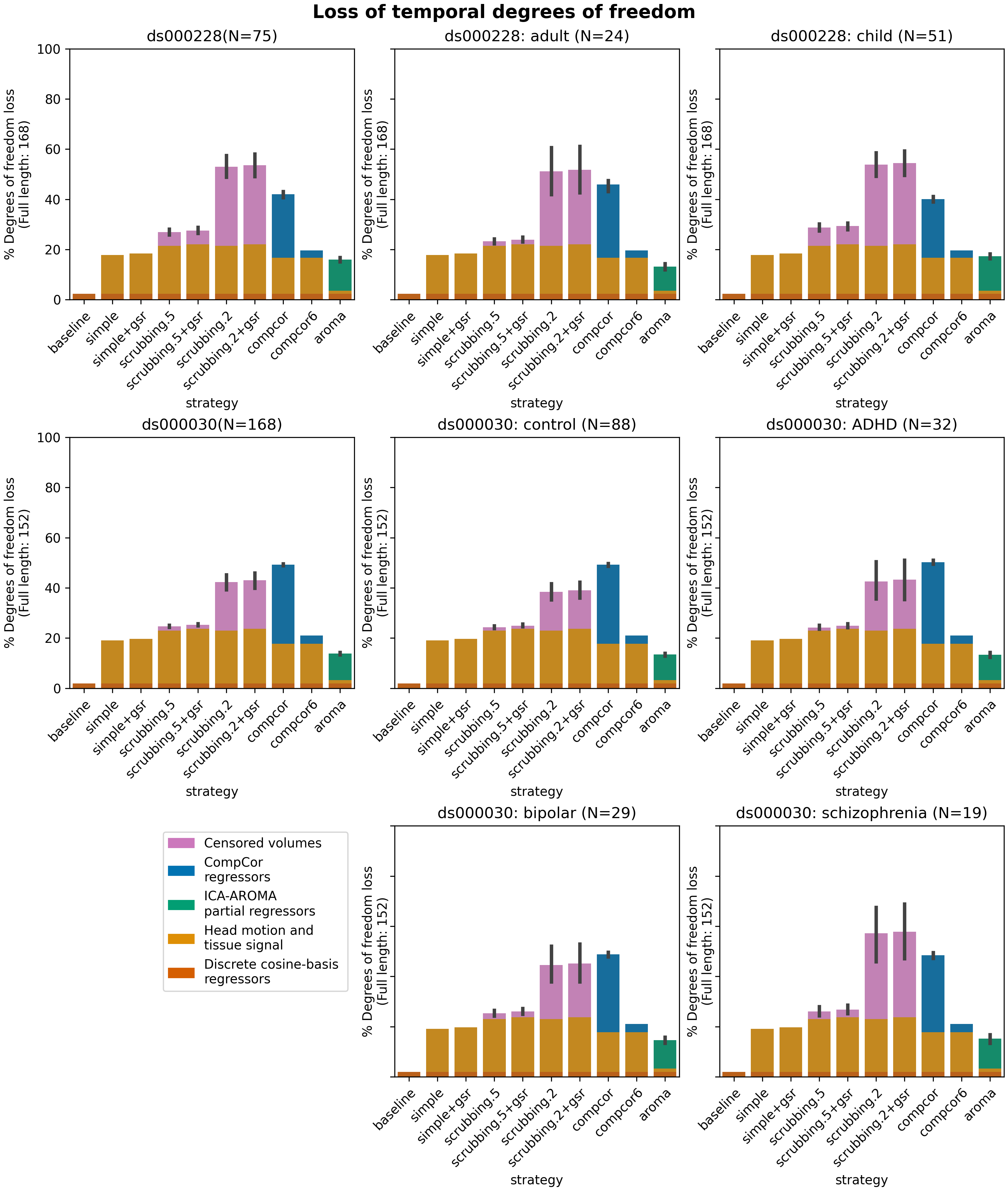

Supplement: S1 Fig — (TIFF) [file pcbi.1011942.s002.tiff]

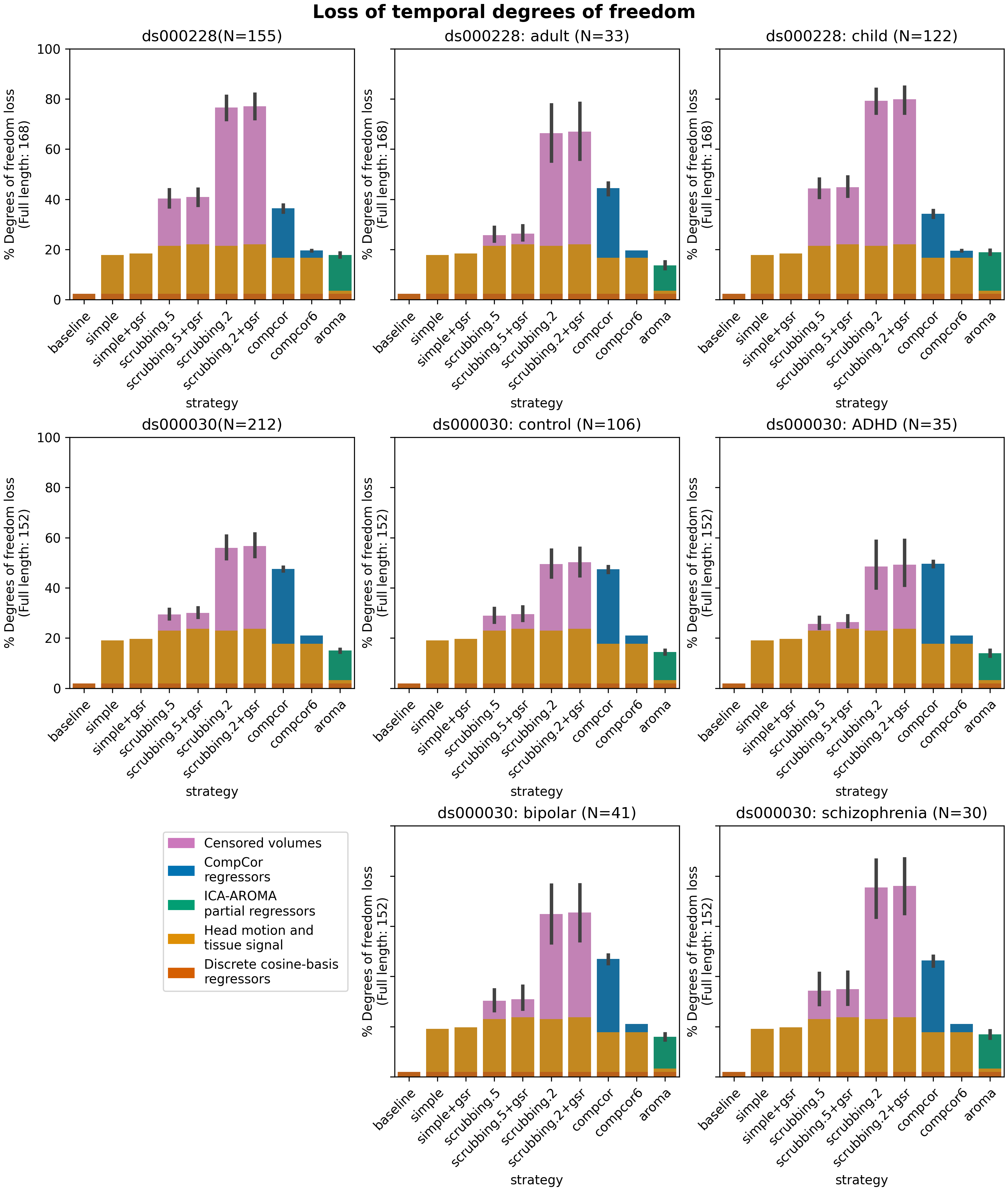

Supplement: S2 Fig — (TIFF) [file pcbi.1011942.s003.tiff]
