# Supplementary material for: Continuous evaluation of denoising strategies in resting-state fMRI connectivity using fMRIPrep and Nilearn
Source: PLoS Comput Biol. 2024 Mar 18;20(3):e1011942. doi: 10.1371/journal.pcbi.1011942 (PMC10977879; doi:10.1371/journal.pcbi.1011942)
Supplement: Attachment — Submitted filename: fmriprep-denoise-benchmark_ploscompbiol-r1_review-response-1.pdf [file pcbi.1011942.s004.pdf]

Response to reviewers

Reviewer #1

The manuscript "Continuous Evaluation of Denoising Strategies in Resting-State fMRI Connectivity Using fMRIPrep and Nilearn" is a very impressive body of work. The analyses employed are very comprehensive and well thought out. Moreover, the authors have gone above and beyond regarding the reproducibility of the available code by sharing an interactive notebook on the neurolibre platform. The companion notebook, means that readers will be able to understand everything about how the analysis was run. Readers can test what could have gone differently with different settings. Even more helpful, due to the completeness (from data loading to results) of the code available - readers are provided with a clear understanding of how to adapt this workflow for use on new datasets. Additionally, this manuscript is presented in concert with the authors' contributions to the heavily adopted, and open-source nilearn python package, which means that their interfaces for loading confound regressors and applying confound regression with scrubbing are expected to be highly adopted by the field. Given the richness of this analysis, a few additional questions/comments struck me while reading this work. Those are listed below.

We would like to thank the reviewer for checking on the Neurolibre preprint and recognise the effort of contributing to existing open source projects.

R1.1. The authors were correct that the loss of degrees of freedom is an important consideration when determining what denoising strategy to use. Those methods with the highest loss of degrees of freedom require more participants to be excluded from the analysis. I understand that the authors needed to exclude participants with high and moderate motion to evaluate the scrubbing.2 strategy on equal footing to the other methods. However, I was struck by how many participants needed to be excluded, particularly from specific groups (i.e. children and participants with schizophrenia). The exclusion of this many participants would result in a severe loss of power, meaning that particular group comparisons are no longer feasible. While it may not be the role of this paper to comment on thresholds for participant exclusion due to motion, I would appreciate it if the number of participants excluded were stated more clearly, perhaps by reporting on the starting n's and % excluded in Table 3.

As suggested, we added the number (n) and percentage (%) of the sample excluded from analysis in Table 3. We agree with this reviewer that the loss in sample size is very large for certain datasets and subgroups. We added the following paragraph in the discussion section on page 26, line 675 - 685:

> *… The current evaluation was performed on datasets after excluding subjects with gross in-scanner motion, as per existing literature [6,7]. The aim of the exclusion allowed evaluation of denoising strategies on the mitigation of artifacts due to micro-movements. However, the exclusion criteria would result in loss of power in downstream analysis in certain demographics, and was particularly apparent here for the "child" group in ds0000228 and the "schizophrenia" group in ds000030. We encourage researchers to*

take this potential loss in sample size into account for selecting an appropriate denoising strategy for their study. Our Neurolibre companion offers comparisons of mean framewise displacement and loss of temporal degrees of freedom at two different motion exclusion thresholds or without exclusions, for reference.

R1.2. Following from comment R1.1 I am also concerned about the possible unequal loss of degrees of freedom across subgroups of interest. For example, do strategies like compcor or scrubbing.2 result in higher losses of degrees of freedom in children than adults? Does this lead to increased downstream differences in data quality? At the very least, perhaps the authors could report the loss of degrees of freedom broken down by group. The authors would also consider a metric for evaluation of strategy: loss of df within the subgroup of particular interest (i.e. patients) or the interaction between groups in loss of df. Should these measures also be reported in the workflow?

Data excluding due to motion, indeed, severely impacts some groups more than the general population. We have now reported the number of participants excluded and addressed the loss of power as a limitation and a factor for researchers to consider when selecting denoising strategies for their study. It is also worth noting that in Ciric et al. 2017, similar motion based subject exclusion criteria was used.

Degrees of freedom broken down by subgroups certainly identify some differences, see the new supplemental information S1 Figure of the revised manuscript. For instance, gentle scrubbing (0.5mm FD cut-off) only leads to substantial loss in degrees of freedom for the “child” group of ds000228. However, despite some quantitative differences, the overall behavior of the respective methods was consistent across datasets and groups.

We made changes to the text in the section “Loss in temporal degrees of freedom varied markedly across strategies and datasets” to reflect these observations.

R1.3. The evaluation of denoising strategies across versions of fMRIprep may be better summarized in a table instead of a list. In other words, rather than saying that results 1, 4, 5 or the above list were also observed in the new analysis - it might have been easier for the reader to see that concept presented in a column (with checkmarks and exes?) beside the original item.

We agree this information is better summarized as a Table, which we added to complement the existing text (see Table 4 on page 22 in the revised manuscript).

R1.4. There is a growing concern in the field that newer, particularly newer multi-band fMRI sequences with shorter TR, higher distortion, and smaller voxel size may have different motion concerns because 1) the higher TR allows for rhythmic breathing-related motion to be captured and 2) because the higher distortions may make it harder to register frames in the motion correction algorithm - leading to artificially reduced estimates of framewise displacement. These potential differences make me wonder if the results of this workflow would be the same if this workflow was applied to a newer dataset with multi-band acquisition parameters. I understand that the incorporation of a whole new dataset may be beyond the scope of the current investigation, but perhaps some words of caution and some

mention of the need for further studies of motion benchmarks across of fMRI sequence parameters may be of merit in the discussion.

We agree this is an important limitation in the current benchmark, and we now address this limitation in the discussion section of the revised manuscript, where we briefly comment on fMRIPrep's multiband preprocessing.

On page 27, line 686 - 693:

> *Unclear implication for datasets with lower TR or multiband scanning sequence: The current benchmark used two datasets with TR of 2 to 2.5 seconds, thus the conclusions are limited to fMRI datasets with a similar scanning sequence. For multi-band fMRI sequences with shorter TR, there may be different motion concerns such as respiratory motion [41,42]. It would be of the community's interest to explore the current workflow on multiband fMRI datasets, and including physiology related denoising strategies and different quality control metrics [43] to address the current limitations.*

Reviewer #2

Thank you for sending me this article for review. The work describes the development and assessment of an API for reproducible denoising as well as a comparison of a broad range of common denoising strategies post fmriprep processing.

This article is excellent work and will be extremely useful for the field. Importantly it provides a comprehensive assessment of several denoising strategies and gives reasonable recommendations for the users. Further, all data and scripts are easily available through the reproducible preprint server NeuroLibre.

Thank you!

My only comments are as follows:
R2.1. on p. 18 "Significance tests associated with the partial correlations were performed. P-values above the threshold of α = 0. 05 were deemed significant." - do the authors mean "above the threshold" or "below the threshold"? Greater or less than 0.05?

Thanks for catching this error! It has now been corrected in the text on page 16 line 417. The code was implemented correctly. See: https://github.com/SIMEXP/fmriprep-denoise-benchmark/blob/b9d44504384b3641dbd1d063105cb6eb99713488/fmriprep_denoise/features/quality_control_connectivity.py#L13-L40 (accessed Jan 15th 2024).

R2.2. Figure 12 is somewhat confusing. I would have interpreted the "Ranking" to be both in each cell in the matrix (as denoted by the size and color of the dots, as well as (and most importantly) by row. The rows do not seem to be ranked. In particular, in the conclusion the authors recommend the simple+gsr strategy, yet this strategy does not fall on the far right of the frame. The image should either be changed or the text should make things clearer

We intentionally kept all the order of the strategies consistent across figures instead of ranking them for two reasons. Firstly the visual display consistency would be helpful when inspecting reports side by side. Secondly we did not want to imply that a single strategy emerged as a clear best approach, as there exists a clear trade-off between denoising quality and loss of degrees of freedom. Our visualization aims at making this trade-off easy to understand across multiple performance metrics, rather than ranking methods overall. We revised the title and caption of revised caption of Figure 12 in order to highlight this nuance. On page 24, line 610 - 616:

> *We ranked strategies across four metrics from best to worst. Larger circles with brighter color represent higher ranking. The order of the presented strategies were kept consistent with the reports from the result sections. Metric "correlation between network modularity and motion" has been excluded from the summary as it is potentially a poor measure. Loss of temporal degrees of freedom is a crucial measure that should be taken into account alongside the metric rankings.* ***A clear trade-off is apparent between loss in degrees of freedom and the quality of denoising, so no overall ranking of methods is derived from this analysis - see text for a summary of key takeaways.***

I look forward to seeing this article in print.

Thank you for the enthusiasm and the constructive feedback.

Reviewer #3

General comments:

This manuscript presents a reproducible denoising benchmark for the evaluation of different research software and denoising strategies. This is a very useful tool, and the manuscript does a very good job at describing its implementation as well as summarizing the benchmark outputs for several denoising strategies, datasets, and software versions. My main concern/suggestion would be to moderate some of the paper's conclusions, as they may be extrapolating beyond the evidence provided by the benchmark results (e.g. point 7 below).

Thank you!

Specific comments:

R3.1, Could you please comment on the scrubbing choice of DVARS threshold (I believe the fMRIPrep default standardized DVARS threshold is 1.5 instead of 3)?

Thank you for raising this point and we would like to clarify what has been done as we believe our original manuscript was unclear.

1. You are correct - the fMRIPrep default is 1.5 and there's a mistake in the default value of the `load_confounds` function. We have opened a pull request to correct the default value. The changes will go through formal depreciation cycles of Nilearn. In version 0.11.0, the user

will see a warning regarding these values and a note that the default value will change in version 0.13.

2. The fMRIPrep default values will make no impact in the current workflow as we did not use the fMRIPrep generated scrubbing one-hot regressors. Instead, the `load_confounds` function creates sample_mask from the framewise displacement and standardized DVARS in the confounds reports. We made that choice because users can easily explore different thresholds without having to run fMRIprep again, but this comes at the cost of redundancy in the two code bases, and potential for differences in implementation.

3. We did not use the DVARS threshold. The aim of this manuscript is to replicate the viable strategies from Ciric et. al., 2017. In their scrubbing implementation (Model 8), framewise displacement only was used for identifying high motion volumes.

Lastly, we understand that this confusion arose from our original presentation of Tables 1 and 2. In Table 1, we showcased the default values of each strategy in the `load_confounds` function in order to explain the implementation details. Table 2 contained the exact parameters used for each strategy and the details for scrubbing, which differed from these defaults values, but did not indicate DVARS threshold (as it was not used at all). To further clarify for readers and address the concern raised by this reviewer, we have added a column for `std_dvars_threshold` in Table 2 and specified the value entered to disable the application (`None`).

R3.2. Please elaborate on the details and rationale of the additional strategies "compcor6" (if I understand correctly "compcor6" represents a choice of 6 components from WM and CSF vs. "compcor" which uses a variable number of components per subject accounting for 50% of the total variance?) and "scrubbing.2" (if I understand correctly it uses a more conservative FD>0.2mm threshold definition?)

The details of implementation for compcor6 and compcor can be found in the method section on page 32 line line 880:

> *We evaluated common confound regression strategies that are possible through fMRIPrep-generated confound regressors ….*

See S1 Annex B for the explanation and original reference of the strategies.

R3.3. The chosen set of pre-defined strategies (simple, scrubbing, compcor, and ica_aroma; with or without global regression) captures well some of the common approaches used by AFNI and DPABI (e.g. doi:10.3389/fnins.2022.1073800 and doi:10.3389/fnins.2023.1069639) but it would perhaps benefit from adding another common approach used by CONN combining scrubbing and aCompCor (e.g. the default settings in the CONN toolbox when importing fMRIPrep data will use motion(n=12), WM(n=5), CSF(n=5), scrubbing(FD>.5); see doi:10.3389/fnins.2023.1092125);

Thank you for bringing this method to our attention. We agree this is a common strategy used in the community, which could have been included in the current benchmark. We however decided against expanding the scope of strategies evaluated in the benchmark for the following reason. The denoising strategies implemented in our benchmark correspond to

those available through `load_confounds_strategy` in the nilearn package. Selecting (and naming) these strategies was an iterative work done during development of the corresponding feature, and involved the larger nilearn developer community rather than just the team involved in this paper. Adding a new strategy would require opening a new pull request, and convincing the developer team to incorporate these changes. The outcome and timeline of this process is unclear. This addition would also require updating every figure in the paper. We believe this request is out of the scope of a minor revision and we ultimately decided against including this method in the manuscript. We did add a paragraph in the method section about this strategy and how it would be valuable to include it for future iterations of the denoising benchmark.

Page 32 line 885 - 887:

> Another excluded approach was commonly used by CONN combining scrubbing and aCompCor [48], because we want to focus on strategies corresponding to `load_confounds_strategy` and past benchmark literature. However users can use `load_confounds` to implement this strategy, as follows:

```
from nilearn.interfaces.fmriprep import load_confounds
confounds_simple, sample_mask = load_confounds(
      fmri_filenames,
      strategy=["high_pass", "motion", "compcor", "scrub"],
      motion="derivatives", scrub=0, fd_threshold=0.5,
      std_dvars_threshold=None, compcor="anat_separated", n_compcor=5)
```

R3.4 In the 'Software implementation' section please describe explicitly the mathematical operation implemented by the denoising procedure (e.g. a linear regression of noise components, performed separately for each individual functional run).

The computation was implemented through linear regression using the function signals.clean from the Nilearn library. We added the following information in the manuscript to clarify how the denoising is implemented:

1. We have clarified the mechanism of Nilearn's NiftiLablesMasker on page 7 line 243 - line 256 :

   … The filtered confounds and the corresponding preprocessed NIFTI images were then passed to the Nilearn masker generated with the atlas where the underlying function nilearn.signals.clean applied the regressors for denoising (see https://nilearn.github.io/stable/modules/generated/nilearn.signal.clean.html).....

2. We added the mathematical equations describing the denoising regression procedure in supplemental information S1 Text Annex E.

R3.*5. Relatedly, please clarify whether scrubbing is implemented as a set of additional*

regressors (one per outlier timepoints) introduced in the denoising linear regression step, or if it is implemented as "censoring" (explicitly removing individual timepoints from the data, before or after other denoising steps). If the latter, I would strongly suggest to add minimally an option to use the former approach, as it would avoid the concern regarding data continuity mentioned in other parts of this manuscript (see point 7 below)

Thanks for the concern. The approach implemented in nilearn.singal.clean is the censoring approach. The regression approach was not considered during the discussion phase when implementing the feature in nilearn, hence not implemented. Critically, in terms of the application of scrubbing to functional connectivity, we do not think there are practical differences between the two approaches.Please see point 7 below.

R3.*6. For the computation of QC-FC correlations, please comment on the choice of controlling by subject's age and sex. It seems counterintuitive that correlations between subject motion and functional connectivity that were caused by, for example, having larger motion in younger participants, would be explicitly disregarded when evaluating the relative success of a denoising strategy. If possible please report also FC-QC values without covariate correction.*

Thank you for the comment. We aimed to replicate the existing work of Ciric et al. (2017) and the metrics were calculated in the same way as the original work. This choice is made explicit in our manuscript.

Page 4 line 139:

> *…The benchmark systematically evaluates the impact of denoising choices using a series of metrics based on past research [6,7].*

The reason age and sex are regressed out in these previous works is precisely because they can impact both motion and functional connectivity. If these two variables were not regressed out from either motion or functional connectivity, the correlation between motion and functional connectivity will be driven by these two variables (a potential case of Simpson's paradox), thus we cannot assess the true correlation between motion and functional connectivity. We hope this addresses the concern.

R3.7. The 'conclusions' section seems to assume that scrubbing is somehow incompatible with analyses that require "continuous sampling time series". I believe this would only be true for a "censoring" approach that explicitly removes individual timepoints from the data, while a more common "regression" approach, that simply uses those individual timepoints as additional regressors either during denoising or during first-level analyses, would preserve the data continuity and avoid this concern. Given that the latter (regression-based) scrubbing approach is highly prevalent in the literature as well as across some of the most common software packages that implement denoising (e.g. AFNI, CONN, DPABI, C-PAC) I would recommend either implementing this approach if not done already (see point 5 above) or reconsidering the paper's strategic recommendations.

The regression approach is equivalent to imputing the high motion time points with the average of the remaining time series. In this work, we rely on fMRI functional connectivity metrics, and the two approaches (regression and exclusion of time points) are equivalent.

That is, correlating time series with censored volumes or replacing censored volumes with time series average leads to the same results. Thus this proposed change will not change the current results. More importantly, the regressor approach is also a form of interruption of continuous time series, and will disrupt many operations such as, e.g., calculating a power spectrum. We added this point in the method section of the manuscript.

Page 32 line 896 - 905:

> *For scrubbing based strategies, the nilearn.signals.clean function censors the high motion time points before denoising with linear regression, known as the censoring approach. We did not use another common approach which is entering the high motion time points as one-hot encoders in the same linear regression with other confound regressors, known as the regression approach. The regression approach is equivalent to imputing the high motion time points with the average of the remaining time series. The benchmark assessed fMRI functional connectivity metrics. The two approaches will produce numerically equivalent results. It's important to note that scrubbing strategy performed with either approach is a form of interruption of continuous time series, and will disrupt many operations such as, e.g., calculating a power spectrum.*

R3.8. The 'conclusions' section would perhaps benefit from some additional thoughts on the potential applications and limitations of the proposed reproducible benchmark when used to help guide strategic recommendations for different denoising procedures or pipelines, as used in the paper. In particular, the results of this paper, consistent with a lot of the literature, indicate that the relative success/failure of different denoising strategies may vary strongly across different datasets (what works best for one dataset may not work for another). From that perspective, I wonder whether the current benchmark could be extended to include additional datasets, or even adapted for researchers to use on their own datasets in order to evaluate the specific merits of different denoising strategies on their own data. I would also love to hear the authors' thoughts on: a) the generalizability of the conclusions drawn from these datasets to others; and b) how this benchmark may be used not only by researchers interested in methodological advancements in denoising procedures but also perhaps by general researchers on their own datasets as part of general quality assurance procedures for functional connectivity analyses.

Thank you for these insightful comments.

Regarding point a, we fully agree that the behavior of denoising methods will depend on the datasets used for evaluation, and a comprehensive benchmark should aim at a wide diversity of technical and biological characteristics. In this work, we aimed at a balance between such a wide scope and practical considerations: using readily available open datasets with clear documentation and also keeping execution time within reason for ease of replication. Yet, there are important datasets we could have included, e.g. people with dementia or multiband fMRI data (see R1.4.). We have added a paragraph in the discussion to discuss the generalisability of the current research object on page 27 line 719 - 728:

> *The current workflow is presented as a research object rather than a software due to the lack of generalizability on other datasets. For the analysis after fMRIPrep, there*

are two practical reasons for this choice. Firstly, compared to a piece of well packaged software, research objects allow more flexibility for changes for development. Secondly and most importantly, creating a clean, generalizable solution will require the data to be standardized. Although fMRIPrep outputs are standardized, the demographic information is coded differently across datasets. Currently the BIDS specifications do not impose restrictions on the label for phenotypic data yet, thus we had to manually harmonize the label for age, gender, and group information. As an alternative, full documentation to re-execute the workflow, from fetching datasets to running the analysis, is available as part of the research object.

We have also toned down the current conclusions to reflect on the potential lack of generalisability (see Abstract, discussion section "Re-executable research object" and Conclusion).

Regarding point b, the aim of this manuscript was to provide recommendations based on a fully reproducible workflow. Many components of this workflow are not meant to be easily reusable, although most of the code is (through the load_confounds_strategy in nilearn). Other components, such as the code used to preprocess the data, require more involved coding. Although we believe it will be straightforward to extend this benchmark to include more datasets in the future, it is not readily available as a feature for the current implementation. We have modified the discussion to reflect on this point on page 27 line 730 - 738:

There are additional benefits to creating a re-executable denoising benchmark. Although the code is not readily designed to process new datasets, it contains good prototypes for what could become different BIDS-apps for post processing [18]: a connectome generation BIDS-app and a denoising metric generation BIDS-app. BIDS-app is easier for user adoption under the BIDS convention and can expand the scope of the benchmark from the two datasets shown here to any BIDS-compliant dataset. The process of creating this benchmark also provides valuable first hand information about runtime, and the impact of atlas choice on computational costs, which we did not cover here but has big practical implications.
